# Analysis of Cadmium Root Retention for Two Contrasting Rice Accessions Suggests an Important Role for *OsHMA2*

**DOI:** 10.3390/plants10040806

**Published:** 2021-04-20

**Authors:** Moez Maghrebi, Elena Baldoni, Giorgio Lucchini, Gianpiero Vigani, Giampiero Valè, Gian Attilio Sacchi, Fabio Francesco Nocito

**Affiliations:** 1 Dipartimento di Scienze Agrarie e Ambientali—Produzione, Territorio, Agroenergia (DISAA), Università degli Studi di Milano, 20133 Milano, Italy; moez.maghrebi@unimi.it (M.M.); giorgio.lucchini@unimi.it (G.L.); gianattilio.sacchi@unimi.it (G.A.S.); 2 Istituto di Biologia e Biotecnologia Agraria (IBBA), Consiglio Nazionale delle Ricerche (CNR), 20133 Milano, Italy; elena.baldoni@cnr.it; 3 Dipartimento di Scienze della Vita e Biologia dei Sistemi, Università degli Studi di Torino, 10135 Torino, Italy; gianpiero.vigani@unito.it; 4 Centro di ricerca per la Cerealicoltura e le Colture Industriali (CREA-CI), 13100 Vercelli, Italy; giampiero.vale@uniupo.it; 5 Dipartimento di Scienze e Innovazione Tecnolgica (DISIT), Università degli Studi del Piemonte Orientale Amedeo Avogadro, 13100 Vercelli, Italy

**Keywords:** *Oryza sativa* L., cadmium, zinc, cadmium translocation, cadmium root retention

## Abstract

Two rice accessions, Capataz and Beirao, contrasting for cadmium (Cd) tolerance and root retention, were exposed to a broad range of Cd concentrations (0.01, 0.1, and 1 μM) and analyzed for their potential capacity to chelate, compartmentalize, and translocate Cd to gain information about the relative contribution of these processes in determining the different pathways of Cd distribution along the plants. In Capataz, Cd root retention increased with the external Cd concentration, while in Beirao it resulted independent of Cd availability and significantly higher than in Capataz at the lowest Cd concentrations analyzed. Analysis of thiol accumulation in the roots revealed that the different amounts of these compounds in Capataz and Beirao, as well as the expression levels of genes involved in phytochelatin biosynthesis and direct Cd sequestration into the vacuoles of the root cells, were not related to the capacity of the accessions to trap the metal into the roots. Interestingly, the relative transcript abundance of *OsHMA2*, a gene controlling root-to-shoot Cd/Zn translocation, was not influenced by Cd exposure in Capataz and progressively increased in Beirao with the external Cd concentration, suggesting that activity of the OsHMA2 transporter may differentially limit root-to-shoot Cd/Zn translocation in Capataz and Beirao.

## 1. Introduction

Cadmium (Cd) is a toxic heavy metal for most living organisms and represents one of the principal dangerous substances released into soil, water, and atmosphere by anthropogenic activities [1,2,3,4]. Although Cd is not essential for plants, it can be easily absorbed by roots and accumulated in different plant organs. Such a relative high mobility of Cd in the soil–plant systems makes plant-derived foods the primary pathway for Cd entry into the agricultural food chain, and thus the major source of Cd exposure for humans [5,6,7].

Rice (*Oryza sativa* L.), an important staple food for more than half of the world’s population, has been recognized as the major source of Cd intake in Japan and other Asian countries, where a high prevalence of renal proximal tubular dysfunctions has been documented [8,9,10]. Rice often accumulates high levels of Cd in grains, reaching values exceeding the limit of 0.4 mg kg^−1^ established by the Codex Alimentarius Commission of FAO/WHO [11]. For these reasons, several efforts are requested to minimize Cd accumulation in rice above–ground tissues both at the agronomic and genetic level, by developing approaches aimed at reducing Cd availability in soils and introducing new rice cultivars with more suitable Cd accumulation profiles [4,12,13,14,15,16].

Although a considerable natural variation in Cd accumulation profiles has been described in several crop species, including rice [14,15,17,18,19,20,21], most of them retain much of the Cd within the roots [5,16,22,23,24,25]. Such a general behavior—thought to be crucial for preserving the photosynthetic apparatus against Cd injury—results from the activity of a complex “firewall system” which, retaining Cd in the root cells, determines the amount of free Cd ions available to be loaded into the xylem and then potentially translocated from root to shoot. Root-to-shoot Cd translocation via the xylem has been described as the main process determining the accumulation of Cd rice in rice shoots and grains [15], and its dependence on both Cd chelation/sequestration and xylem loading has been largely described [24,25,26,27].

Most of the Cd ions taken up by plants are trapped inside the roots through mechanisms involving Cd selective binding to molecules or cell sites with high affinity for the metal, or Cd transfer across a membrane into an intracellular compartment [28]. Phytochelatins (PCs) and related thiols have been described as the principal Cd chelators in the root cells. They are a class of cysteine-rich peptides, non-translationally synthesized from glutathione (GSH) in a transpeptidation reaction catalyzed by the enzyme PC synthase (PCS) [29,30,31,32]. PCs form complexes with Cd that can be compartmentalized into the vacuoles, and thus retained within the root cells. Although Cd is largely bound by PCs and other thiols [25], a possible contribution of these molecules in determining the intraspecific differences in Cd root retention remains to be elucidated in rice. Part of the free Cd ions inside the root cells can be directly compartmentalized into the cell vacuoles through the activity of the P_1B_-type ATPase OsHMA3, which moves the free Cd ions into the vacuoles. Direct vacuolar sequestration has been clearly shown as one of the main determinants explaining variation in Cd systemic allocation in rice [16]. Only the free Cd ions escaping the root firewall system can be considered potentially available to be translocated via the xylem in a root-to-shoot direction.

It has been shown that Cd partially competes with zinc (Zn) for accumulation in the shoots, as they probably use the same transport systems to be loaded into the xylem [27]. A promising candidate protein for such an activity in rice is the P_1B_-type ATPase OsHMA2, which was found to be expressed in the vascular bundles of roots, where it likely contributes to mediate both Cd and Zn translocation [25,33,34]. Loss of *OsHMA2* function in insertion mutants decreased the root-to-shoot translocation ratio for both Cd and Zn, as well as Cd accumulation in leaf and grains [33,34]. Finally, an involvement of OsHMA2 in the preferential distribution of Cd and Zn to the developing tissues of rice has been hypothesized by Yamaji and coworkers [35].

Selection of low-Cd accumulator rice accessions is still the most hopeful approach to minimize the dietary intake of Cd, in terms of both effectiveness and economic value [14,25]. In this paper, we describe and compare two rice accessions differing for their capacity to retain Cd within the roots, with the specific aim of dissecting the relative contribution of each element of the root firewall system in determining Cd systemic distribution between roots and shoots.

## 2. Results

This paper describes and compares two rice accessions, Capataz and Beirao, selected as the most contrasting accessions following a preliminary screening for different traits related to Cd accumulation and tolerance performed on a *japonica* rice panel.

### 2.1. Plant Growth under Cd Stress and Cd Partitioning between Roots and Shoots

Rice plants were grown hydroponically and exposed for 10 days to a broad range of Cd concentrations (from 0.01 to 1 μM) in the growing medium.

**Figure 1:**
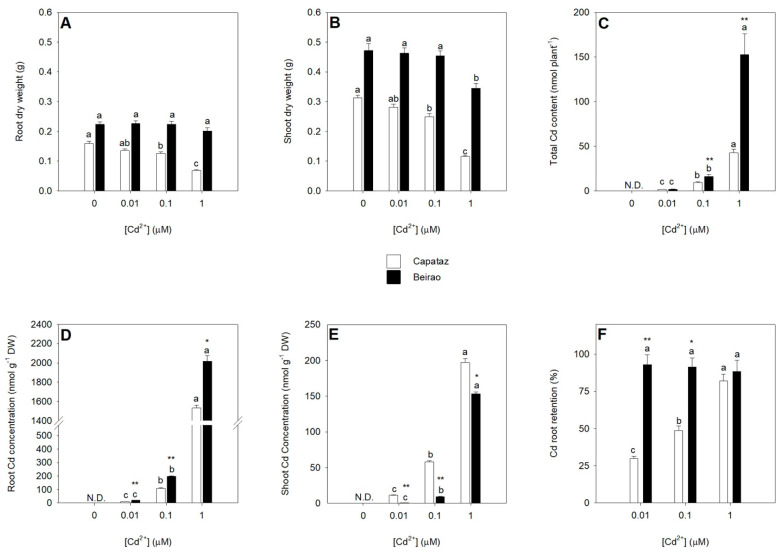
Plant growth under Cd stress and Cd partitioning between roots and shoots. Capataz (white bars) and Beirao (black bars) were exposed to different Cd concentrations (0.01, 0.1, and 1 µM) for 10 days. Root (**A**) and shoot (**B**) dry weight. Total Cd content in a whole plant (**C**). Cd concentration in roots (**D**) and shoots (**E**). Cd root retention (**F**). Bars and error bars are means and SE of two experiments run in triplicate (n = 6). Different letters indicate significant differences among treatments (*p* < 0.05). Asterisks indicate significant differences between Capataz and Beirao (Student’s *t*-test; * 0.001 ≤ *p* < 0.005; ** *p* < 0.001). DW, dry weight.

Results showed that Cd exposure significantly inhibited the growth of Capataz. Growth inhibition, calculated with respect to the control (absence of Cd), progressively increased from 14.7 to 56.9% and from 4.7 to 63.1%, in roots and shoots, respectively, as Cd external concentration did. On the other hand, in the same conditions, Cd did not produce significant effects on the growth of Beirao, except at the highest concentration analyzed (1 μM), where a significant inhibition (26.8%) of shoot growth was observed (Figure 1A,B).

The total amount of Cd absorbed by plants increased by enhancing the external concentration of the metal. Except for the lowest Cd concentration analyzed, the amount of the metal in the whole plants was significantly higher in Beirao than in Capataz (Figure 1C). Cd concentrations in root tissues were significantly higher in Beirao than in Capataz and increased as Cd concentration in the external medium did (Figure 1D). On the contrary, Cd concentration in the shoots was significantly higher in Capataz than in Beirao, and increased in both the accessions with the external Cd concentration (Figure 1E).

From these data, we can easily evince that the two accessions were characterized by two contrasting behaviors concerning their root capacity to retain Cd (i.e., the percentage of the total Cd retained in the roots): in Capataz, Cd root retention was not constant and rose from 29.8 to 82.2%, while in Beirao, Cd root retention was significantly higher than in Capataz at the lowest Cd concentrations analyzed (0.01 and 0.1 μM) and resulted independent of the amount of Cd in the growing medium (Figure 1F).

### 2.2. Effects of Cd Exposure on Thiol Accumulation

**Figure 2:**
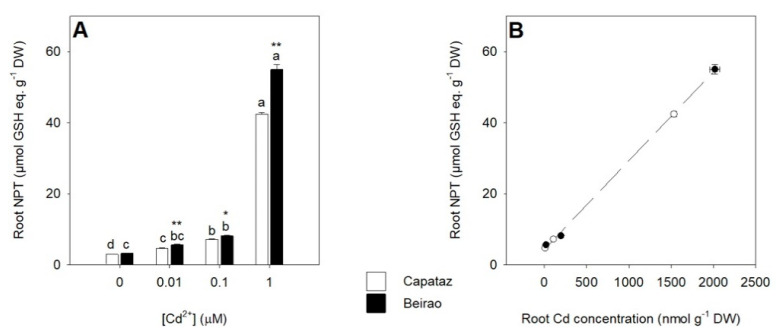
Effect of Cd exposure on non-protein thiol (NPT) accumulation in the roots. Capataz and Beirao were exposed to different Cd concentrations (0.01, 0.1, and 1 µM) for 10 days. NPT concentrations in the roots of Capataz (white bars) and Beirao (black bars) expressed as GSH equivalents (**A**). NPT concentration as a function of Cd concentration in the roots of Capataz (white circles) and Beirao (black circles) (**B**). Data are means and SE of two experiments run in triplicate (n = 6). Different letters indicate significant differences among treatments (*p* < 0.05). Asterisks indicate significant differences between Capataz and Beirao (Student’s *t*-test; * 0.001 ≤ *p* < 0.005; ** *p* < 0.001). DW, dry weight.

Since thiol metabolism may be directly involved in Cd sequestration inside the vacuoles of the root cells, we analyzed the effects of increasing Cd exposure on the accumulation of non-protein thiols (NPTs) in the roots. As expected, NPT levels of the roots increased as Cd concentration in the external medium did and resulted significantly higher in Beirao than in Capataz under all the Cd concentrations analyzed (Figure 2A). The overall relationship between NPT and Cd concentration in the roots of the two rice accessions are reported in Figure 2B.

### 2.3. Effects of Cd Exposure on the Relative Expression of Genes Involved in Cd Chelation, Compartmentalization, and Translocation

**Figure 3:**
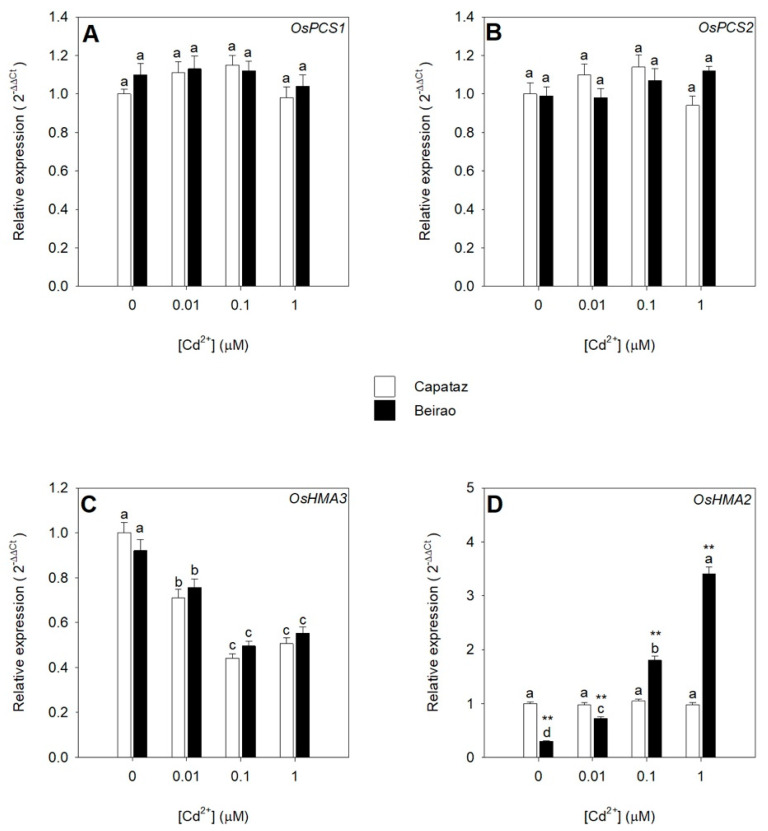
Effects of Cd exposure on the relative transcript abundance of four genes expressed in the roots and involved in Cd root retention. Capataz (white bars) and Beirao (black bars) were exposed to different Cd concentrations (0.01, 0.1, and 1 µM) for 10 days. qRT-PCR was performed on total RNA extracted from the root tissues. *OsPCS1*, *Phytochelatin synthase 1* (**A**). *OsPCS2*, *Phytochelatin synthase 2* (**B**). *OsHMA3*, *Heavy metal ATPase 3* (**C**). *OsHMA2*, *Heavy metal ATPase 2* (**D**). Bars and error bars are means and SE of two experiments run in triplicate (n = 6). Different letters indicate significant differences among treatments (*p* < 0.05). Asterisks indicate significant differences between Capataz and Beirao (Student’s *t*-test; ** *p* < 0.001).

In order to analyze the effect of Cd on the transcripts of the main genes involved in Cd root retention, a qRT-PCR analysis was performed on total RNA extracted from the root tissues. The steady-state level of the transcripts of *OsPCS1* and *OsPCS2*, the two rice genes encoding PC synthases expressed in the roots, was not significantly affected by Cd exposure in both Capataz and Beirao (Figure 3A,B). A different behavior was observed for *OsHMA3*, a gene involved in Cd/Zn sequestration into the vacuoles, whose relative transcript levels decreased as Cd concentration in the external medium increased (Figure 3C). Additionally, in this case, no significant differences were found between Capataz and Beirao. Interestingly, in Capataz, the relative transcript abundance of *OsHMA2*—a gene involved in controlling root-to-shoot Cd/Zn translocation—was not influenced by Cd exposure, while in Beirao, it progressively increased as the Cd concentration in the external medium did (Figure 3D). It is noteworthy that in the absence of Cd, the amount of *OsHMA2* transcript was significantly lower in Beirao than in Capataz.

### 2.4. Effects of Cd on Zn Partitioning between Roots and Shoots

**Figure 4:**
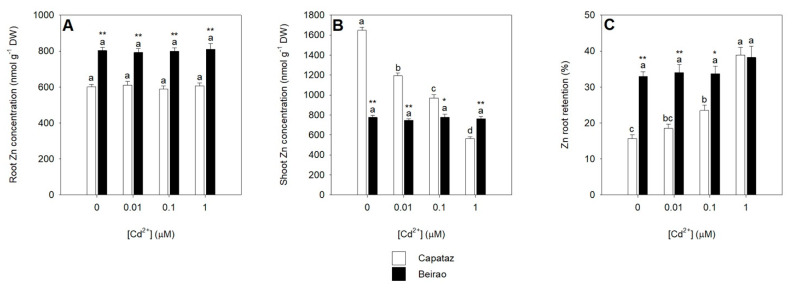
Effects of Cd on Zn partitioning between roots and shoots. Capataz (white bars) and Beirao (black bars) were exposed to different Cd concentrations (0.01, 0.1, and 1 µM) for 10 days. Zn concentration in roots (**A**) and shoots (**B**). Zn root retention (**C**). Bars and error bars are means and SE of two experiments run in triplicate (n = 6). Different letters indicate significant differences among treatments (*p* < 0.05). Asterisks indicate significant differences between Capataz and Beirao (Student’s *t*-test; * 0.001 ≤ *p* < 0.005; ** *p* < 0.001). DW, dry weight.

Since root-to-shoot Cd translocation may involve Zn transporters, we analyzed the effects of the different Cd concentrations on Zn partitioning between roots and shoots and root-to-shoot Zn translocation. Results showed that, in the absence of Cd, the Zn concentration in roots and shoots was significantly higher and lower, respectively, in Beirao than in Capataz (Figure 4A,B). Increasing Cd concentration did not significantly affect the Zn concentration in the roots of both the accessions (Figure 4A). On the other hand, the concentration of Zn in the shoots of Capataz significantly decreased as Cd availability in the external medium increased, while in Beirao shoot Zn accumulation was not affected by Cd exposure (Figure 4B). Similarly to what was described in the case of Cd, the capacity of Beirao to retain Zn in the roots was non influenced by Cd, while in Capataz, it significantly increased as Cd external concentration did (Figure 4C).

### 2.5. Analysis of Root-to-Shoot Cd/Zn Translocation

The systemic movement of Cd and Zn from root to shoot was studied by measuring the concentrations of the metals in the xylem sap of Capataz and Beirao plants exposed to different Cd concentrations. In particular, the translocation activities were estimated by determining the amount of Zn and Cd ions loaded and transported in the xylem sap during a 30 min period.

**Figure 5:**
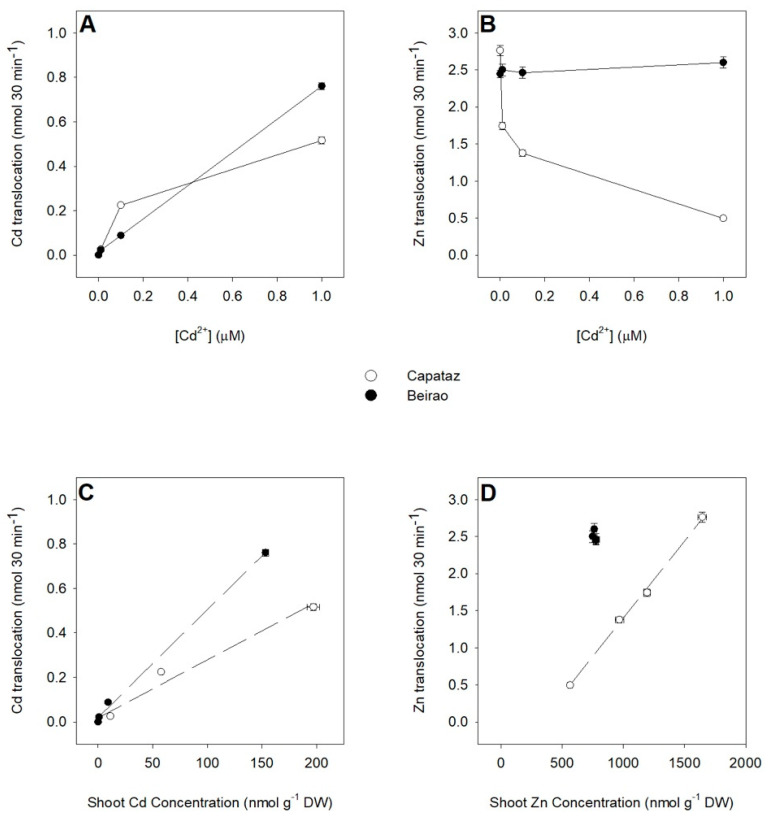
Cd and Zn xylem loading under Cd exposure. Capataz (white circles) and Beirao (black circles) were exposed to different Cd concentrations (0.01, 0.1, and 1 µM) for 10 days. At the end of the exposure period, shoots were separated from roots and the xylem sap exuded from the cut (root side) surface was collected over a 30 min period. Cd ions loaded and transported in the xylem sap (**A**). Zn ions loaded and transported in the xylem sap (**B**). Relationship between Cd ions loaded in the xylem sap and Cd concentration in shoots (**C**). Relationship between Zn ions loaded in the xylem sap and Zn concentration in shoots (**D**). Data are means and SE of two experiments run in triplicate (n = 6). DW, dry weight.

Results showed that in both Capataz and Beirao, increases in Cd external concentration significantly enhanced the amount of Cd ions loaded into the xylem. In Capataz, the Cd xylem loading activity started to approach saturation at 0.1 μM Cd^2+^, while in Beirao, it linearly increased with the external Cd concentration (Figure 5A). On the other hand, Zn xylem loading was progressively lowered by Cd exposure in Capataz but not in Beirao, in which Zn xylem loading was independent of Cd external concentration (Figure 5B). The relationships between Cd and Zn xylem loading and Cd and Zn concentration in the shoots are reported in Figure 5C,D.

## 3. Discussion

In previous works, we showed that the ability of rice to retain much of the Cd taken up within the roots is affected by several factors [25,27]. Such a feature is thought to be essential to prevent excessive Cd accumulation into the shoot, preserving the photosynthetic apparatus against Cd injury. In this paper, we characterized two contrasting rice accessions (Capataz and Beirao) differing for their capacity to retain Cd in the roots. Since Cd root retention capacity generally depends on the amount of Cd available on the growing medium [25], we chose to perform experiments by exposing the rice accessions to a broad range of relatively low and environmentally realistic Cd concentrations.

Data analysis reveals that the two accessions not only differ for Cd root retention—which resulted higher in Beirao than in Capataz—but also for their capacity to accumulate and tolerate Cd (Figure 1). In particular, Cd tolerance was not related to the total amount of Cd absorbed by plants since it resulted higher in Beirao, the most tolerant rice accession used in this work. Thus, at least in our conditions, the differences between the two accessions in their capacity to absorb Cd does not explain the differences observed in their Cd tolerance. Compared with Capataz, the higher Cd tolerance of Beirao was instead related to its higher capacity to retain Cd within the roots, according to several works reporting that Cd tolerance and Cd root-to-shoot translocation are often related in various plant species [36,37,38]. Finally, it is noteworthy that in Capataz, the capacity to retain Cd within the roots depended on the external Cd concentration, while in Beirao, it did not change in all the conditions analyzed (Figure 1F). Such findings strongly suggest that the different distribution of Cd between roots and shoots may depend on differential effects of Cd on the main systems involved in controlling Cd sequestration within the root cells and Cd root-to-shoot translocation.

It is largely accepted that the vacuoles of the root cells are the major sites of Cd sequestration in the free or chelated form [16,32,39,40]. Several studies revealed that most of Cd retained in the roots is immobilized in complexes containing PCs and related thiol compounds [25,27,41,42]. Such ligands—directly derived from GSH—form complexes with Cd that accumulate into the vacuoles [28,30,32]. Since the activation of metabolic detoxification pathways based on the biosynthesis of thiols allows to retain Cd within the roots, we analyzed the effects of Cd exposure on NPT accumulation in the roots of the two rice accessions (Figure 2). Data analysis reveals that for each Cd concentration analyzed, the level of NPTs of the roots was significantly higher in Beirao than in Capataz. However, considering the ratios between NPT and Cd concentration, no significant differences were found comparing the two accessions (Figure 2B). Such a finding suggests that the different pathways observed for thiol accumulation in the roots could be the result of a different Cd accumulation and not the primary cause of the accessions’ specific capacity to retain Cd within the roots. Similar conclusions can be done by analyzing the effects of Cd exposure on the expression levels of *OsPCS1* and *OsPCS2* (Figure 3A,B)—the two main genes involved in PC biosynthesis—since the steady-state level of the two transcripts appeared similar in the two rice accessions and not affected by the external Cd concentrations, as previously reported in other papers [25].

*OsHMA3* has been described as the main candidate for direct Cd sequestration into the vacuoles. Mutations in the sequence of this gene reduce Cd root retention and increase root-to-shot Cd translocation, promoting Cd accumulation in the shoot [16,40]. In our experimental conditions, rising Cd exposures negatively influenced the accumulation of *OsHMA3* transcript in the roots but did not produce differential effects in the two rice accessions (Figure 3C). Such a finding suggests that: (i) the potential ability of the roots of both the rice accessions to retain Cd inside the vacuoles progressively decreased as Cd concentration in the external medium increased; (ii) the different Cd root retention observed in Capataz and Beirao was not ascribable to a differential expression of *OsHMA3*. Moreover, considering that our data are limited to the study of the *OsHMA3* transcript accumulation under different Cd exposure, we cannot definitively exclude that the differences observed in Cd root retention may partially be ascribable to the presence of *OsHMA3* alleles with different functionality, as previously documented in other works [16,40,43,44,45]. However, if this were the case, the expression of hypothetical alleles with different strengths should result in different ratios between NPT and Cd concentration in the two accessions, since OsHMA3 should directly pump Cd into the vacuole.

Several works indicate OsHMA2—a P1B-type ATPase—as the main transporter controlling Zn and Cd translocation in rice [25,33,34]. Our experimental evidence revealed that the expression patterns of *OsHMA2* were differentially affected by Cd exposure in Capataz and Beirao (Figure 3D), suggesting that differences in *OsHMA2* regulation may be involved in determining the differential Cd root retention observed in the two accessions. As previously shown, OsHMA2 can be considered a pivotal element of the firewall system controlling Cd root retention in rice. Indeed, the amount of Cd retained in the roots not only results from a complex equilibrium among the “active” components directly involved in Cd chelation and vacuolar compartmentalization (e.g., PCs and OsHMA3 activity), but also on the activity of transport systems, like OsHMA2, which continuously removing free Cd ions from the root cells, determine the “passive” capacity of the roots to retain the metal ions within them [15,25]. Data here presented showed that the two rice accessions kept constant the Cd/thiol ratio in all the conditions analyzed and progressively reduced their potential ability to directly compartmentalize free Cd ions into the vacuoles of the root cells by reducing *OsHMA3* gene expression. In such a context, it would be expected that the amount of Cd ions escaping the active components of the root firewall progressively increases with the Cd concentration in the root tissues, according to the pictures previously described by fractioning Cd ions accumulated in rice roots [25,27]. Considering the different accumulation patterns of the *OsHMA2* transcripts in the roots, we can reasonably suppose that the activity of the OsHMA2 transporter may differentially limit root-to-shoot Cd translocation in Capataz and Beirao, determining a different Cd root retention. Such a hypothesis seems to be supported by the observation that in Capataz, Cd xylem loading early approached saturation at 0.1 μM Cd^2+^, while in Beirao, it linearly increased with the external Cd concentration (Figure 5A). Moreover, increasing Cd concentration strongly decreased Zn xylem loading in Capataz as possible consequence of different Cd/Zn competitions for the same transport system (Figure 5B). Such an effect was not observed in Beirao, in which Zn xylem loading was independent of Cd external concentration, probably as the result of the progressive accumulation of the *OsHMA2* transcript in the root tissues witch, in turn, enhances both Cd and Zn translocation, reducing at the same time the apparent competition between the two metal ions for the OsHMA2 transport system (Figure 5B). Finally, the differences in the *OsHMA2* expression may also account for the different distribution of Zn between roots and shoots observed in the two rice accessions in the absence of Cd, suggesting that genotypic variations in the regulation of Zn homeostasis and partitioning may also play a pivotal role in determining Cd distribution and tolerance (Figure 4). Under control condition (0 μM Cd^2+^), the concentration of Zn in the shoots was significantly lower in Beirao than in Capataz. Thus, the Cd-induced accumulation of the *OsHMA2* transcripts observed in Beirao could be the consequence of its lower capacity to maintain Zn homeostasis in the shoots under Cd stress.

Taken as a whole data presented in this work confirm that the ability of rice to retain much of the Cd absorbed within the roots results from an equilibrium among the physiological and biochemical processes acting in Cd chelation, sequestration, and translocation. Although phenotypic differences among the rice accessions may mainly depend on specific differences in their capability to compartmentalize Cd into vacuoles of the root cells [16,40,43,44,45], xylem mediated Cd root-to-shoot translocation via OsHMA2 seems to be the main process accounting for the different Cd root retention and tolerance observed in Capataz and Beirao. Since Cd and Zn partially share the same pathways for root-to-shoot translocation, variations in Zn translocation or partitioning efficiency may affect Cd root retention. Thus, the transcriptional response of *OsHMA2* observed in Beirao under Cd exposure could be the result of a complex interaction among a plethora of transporters involved in maintaining Zn homeostasis, rather than the molecular phenotype of an accession-specific *OsHMA2* allele.

## 4. Materials and Methods

### 4.1. Plant Material, Growth Conditions, and Sampling

All the experiments were performed using two rice (*Oryza sativa* L. ssp. *japonica*) accessions, Capataz and Beirao, belonging to a *japonica* rice panel maintained at the CREA-Research Centre for Cereal and Industrial Crops in Vercelli, Italy [46,47].

Rice caryopses were placed on filter paper saturated with distilled water and incubated in the dark at 26 °C. Seven days later, seedlings were transplanted into 3 L plastic tanks (21 seedlings per tank) containing the following complete nutrient solution: 1.5 mM KNO_3_, 1 mM Ca(NO_3_)_2_, 500 μM MgSO_4_, 250 μM NH_4_H_2_PO_4_, 25 μM Fe-tartrate, 46 μM H_3_BO_3_, 9 μM MnCl_2_, 0.8 μM ZnSO_4_, 0.3 μM CuSO_4_, 0.1 μM (NH_4_)_6_Mo_7_O_24_, 30 μM Na_2_O_3_Si (pH 6.5). Seedlings were kept for 12 days in a growth chamber maintained at 26 °C and 80% relative humidity during the 16 h light period and at 22 °C and 70% relative humidity during the 8 h dark period. Photosynthetic photon flux density was 400 µmol m^−2^ s^−1^. At the end of this period, plants were treated with Cd by supplementing the nutrient solutions with different amounts of CdCl_2_ to reach the final concentrations of 0.01, 0.1, and 1 μM. The treatment period was 10 day long. All hydroponic solutions were renewed daily to minimize nutrient depletion.

Plants were harvested and roots were washed for 10 min in ice-cold 5 mM CaCl_2_ solution to displace extracellular Cd [48], rinsed in distilled water, and gently blotted with paper towels. Shoots were separated from roots, and the tissues were frozen in liquid N_2_ and stored at −80 °C or analyzed immediately.

### 4.2. Determination of Cd, Zn and Total Non-Protein Thiols (NPTs)

Samples of c.a. 400 mg fresh weight (FW) of both roots and shoots were mineralized at 120 °C in 5 mL 14.4 M HNO**_3_**, clarified with 1.5 mL 33% (*w*:*v*) H_2_O_2_, and finally dried at 80 °C. The mineralized material was dissolved in 5 mL 0.1 M HNO**_3_** and filtered on a 0.45 μm nylon membrane. Cd and Zn content was measured by inductively coupled plasma-mass spectrometry (ICP–MS; Bruker AURORA M90 ICP–MS).

Total non-protein thiols of roots were determined as described by Nocito et al. [25]. Results were expressed as nanomoles of GSH equivalents.

### 4.3. Analysis of Root-to-Shoot Cd Translocation

At the end of the exposure period, shoots were cut 2 cm above the roots with a microtome blade. Xylem sap exuded from the lower cut surface was collected by trapping into a 1.5 mL plastic vial filled with a small piece of cotton for 30 min. The amount of collected sap was determined by weighing, and the Cd and Zn concentrations were measured by ICP-MS after complete mineralization.

### 4.4. RNA Extraction and qRT-PCR Analysis

Total RNA was extracted from roots using TRIzol^®^ Reagent (Life Technologies, Carlsbad, CA, USA) and then purified using PureLink^®^ RNA Mini Kit (Life Technologies), according to the manufacturer’s instructions. Contaminant DNA was removed on-column using PureLink^®^ DNase (Life Technologies). First-strand cDNA synthesis was carried out using the SuperScript^TM^ III First-Strand Synthesis SuperMix for qRT-PCR (Invitrogen, Carlsbad, CA, USA), according to the manufacturer’s instructions.

qRT-PCR analysis of *OsPCS1* (*Phytochelatin synthase 1*; LOC_Os05g34290), *OsPCS2* (*Phytochelatin synthase 2*; LOC_Os06g01260), *OsHMA3* (*Heavy metal ATPase 3*; LOC_Os07g12900), and *OsHMA2* (*Heavy metal ATPase 2*; LOC_Os06g48720) was performed on first-strand cDNA in a 20 μL reaction mixture containing GoTaq^®^ qPCR Master Mix (Promega, Madison, WI, USA) and the specific primers, using an ABI 7300 Real-Time PCR system (Applied Biosystems, Foster City, CA, USA). The relative transcript level of each gene was calculated by the 2^−ΔΔCt^ method [49] using the expression of the Os*S16* coding for the 40S ribosomal protein S16 (LOC_Os11g03400) gene as reference. Primers for qRT-PCR are listed in Table S1.

### 4.5. Statistical Analysis

Quantitative values are presented as mean ± standard error of the mean (SE) of two independent experiments run in triplicate (*n* = 6). In each independent experiment, three distinct 3 L tanks were used for each control (0 μM Cd^2+^) and Cd treatment (0.01, 0.1, 1 μM Cd^2+^). ANOVA was carried out using SigmaPlot for Windows version 11.0 (Systat Software, Inc., San Jose, CA, USA). Significance values were adjusted for multiple comparisons using the Bonferroni correction. Student’s *t*-test was used to assess the significance of the observed differences between Capataz and Beirao. In both cases, statistical significance was at *p* < 0.05.

## Data Availability

The raw data supporting the conclusions of this article will be made available by the authors, without undue reservation, to any qualified researcher.

## Supplementary Materials

The following are available online at https://www.mdpi.com/article/10.3390/plants10040806/s1, Table S1: Primers used for qRT-PCR analysis.
