# Supplementary material for: Analysis of Cadmium Root Retention for Two Contrasting Rice Accessions Suggests an Important Role for OsHMA2
Source: Plants (Basel). 2021 Apr 20;10(4):806. doi: 10.3390/plants10040806 (PMC8073749; doi:10.3390/plants10040806)
Supplement: Supplementary file 1 [file plants-10-00806-s001.zip › plants-1196579-supplementary.pdf]

Table S1. Primers used for qRT-PCR analysis.

**Gene**	Primer name	Sequence
OsPCS1	OsPCS1F	GAAAGCTTTTGCCGCAATG
	OsPCS1R	CCGCCTGGTTGCTTTCC
OsPCS2	OsPCS2F	TTCGACGAGTCCATGCTTGAC
	OsPCS2R	ATGCCCTCCGCCTTCAC
OsHMA3	OsHMA3F	TCCATCCAACCAAACCCGGAAA
	OsHMA3R	TGCCAATGTCCTTCTGTTCCCA
OsHMA2	OsHMA2F	TGGGTCTCGAGCGTTGAGA
	OsHMA2R	GAGCATAATCAACCAGGACAGATG
S16	S16F	ACGTCGACGAGGCATCCA
	S16R	CGCGACCACCGAACTTCTT
